# Favorable response to PD-1 inhibitor plus chemotherapy as first-line treatment for metastatic gastric mixed neuroendocrine-non-neuroendocrine tumor: a case report

**DOI:** 10.3389/fphar.2024.1295134

**Published:** 2024-02-01

**Authors:** Lingnan Zheng, Lingqi Sun, Ji Ma

**Affiliations:** ^1^ Abdominal Oncology Ward, Division of Medical Oncology, Cancer Center, West China Hospital, Sichuan University, Chengdu, China; ^2^ Department of Neurology, The Air Force Hospital of Western Theater Command, Chengdu, Sichuan, China

**Keywords:** gastric MiNEN, immunotherapy, immune checkpoint inhibitors, PD-L1, chemotherapy

## Abstract

Gastric mixed neuroendocrine-non-neuroendocrine tumor (MiNEN), a rare malignancy, currently has no standard treatment. Here, we report a patient with pathologically confirmed gastric MiNEN following radical surgery with rapid postoperative distant tumor recurrence. Immunofluorescence results suggested intensive lymphocyte infiltration in the tumor. The programmed death receptor ligand 1 (PD-L1) immunohistochemistry 22C3 pharmDx assay showed tumor proportion score was 5% and combined positive score was 10. After 6 cycles of treatment with etoposide and cisplatin in combination with toripalimab, efficacy was assessed as a complete response. Our report shows that for gastric MiNEN patients with high expression of PD-L1, chemotherapy combined with immune checkpoint inhibitors may achieve more significant efficacy.

## Introduction

Mixed neuroendocrine-non-neuroendocrine tumor (MiNEN) is a rare malignancy and has been found in a variety of organs. Gastric MiNEN accounts for less than 1% of all gastric cancers and is characterized by aggressive tumor behavior, high invasiveness, extensive lymph node dissemination and a poor prognosis (Ramos et al., 2021). However, due to the rarity of the disease, there is currently no standard treatment. Here, we report for the first time a gastric MiNEN patient with high expression of programmed death receptor ligand 1 (PD-L1) who rapidly developed disease recurrence and metastasis after radical surgery. The patient received first-line treatment with chemotherapy combined with toripalimab, a monoclonal antibody against human programmed death-1 (PD-1), and the lesions completely disappeared after 6 cycles of treatment without any obvious side effects.

## Case report

**FIGURE 1:**
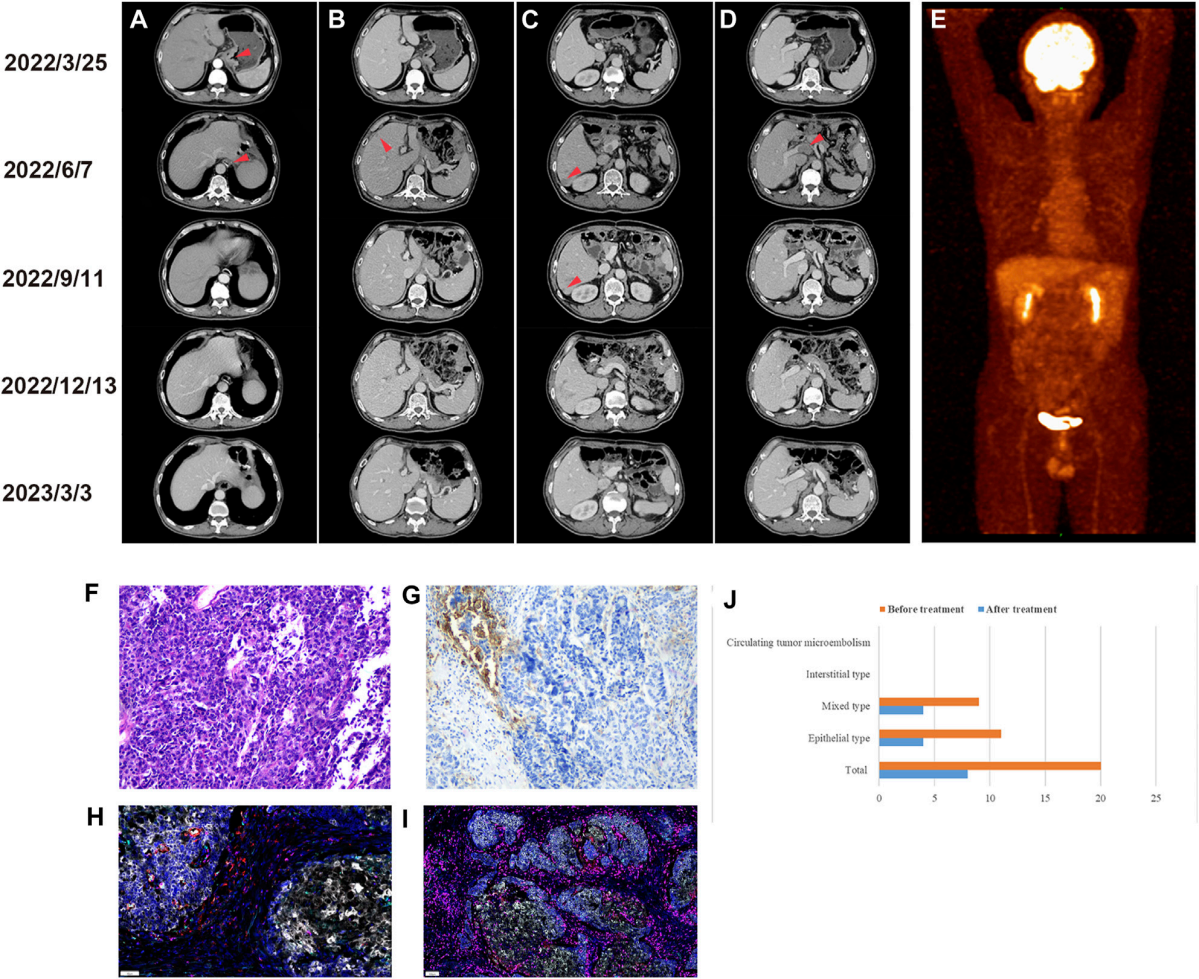
Imaging examination before and after treatment. **(A)** Preoperative CT images of cardiac lesions and postoperative anastomosis. **(B)** Preoperative and postoperative CT images of the left liver lobe **(C)** right liver lobe and **(D)** portal vein. **(E)** Positron emission tomography/computed tomography scan after six cycles of treatment. Preoperative examination (2022/3/25), postoperative examination (2022/6/7), after 3 cycles of treatment (2022/9/11), after 6 cycles of treatment (2022/12/13), last follow-up (2023/3/3). Immunohistochemical staining of gastric MiNEN. **(F)** Hematoxylin and eosin staining. **(G)** Immunohistochemistry of PD-L1. Multiple immunofluorescences of gastric MiNEN. Analysis of the tumor microenvironment including PD-1, PD-L1, T cells, tumor-associated macrophages, and natural killer cells was performed. The merging of fluorescence signals is shown. **(H)** Representative immunofluorescence staining for PD-1 (green), PD-L1 (yellow), CD8 (pink), CD68 (cyan) and CD163 (red). **(I)** Representative immunofluorescence staining for CD3 (pink), CD4 (red), CD20 (green), CD56 (cyan) and FoxP3 (yellow). **(J)** Changes in circulating tumor cells before and after treatment.

In March 2022, a 64-year-old man presented to our hospital with abdominal pain. Gastroscopy revealed a neophyte in the cardia, followed by a biopsy indicating poorly differentiated adenocarcinoma. An abdominal computed tomography (CT) scan showed uneven thickening of the cardia (Figure 1A). The patient subsequently underwent a radical total gastrectomy. Postoperative pathology suggested that the macroscopic type was Borrmann III. The tumor invaded the subserous connective tissue, without involving the visceral peritoneum. The histological diagnosis was MiNEN (Figure 1F), of which approximately 50% were large cell neuroendocrine carcinomas, 45% were poorly differentiated adenocarcinomas, and approximately 5% were squamous cell carcinomas. Moreover, nineteen lymph nodes were examined, with one node showing lymphatic metastasis and another node showing a gastrointestinal stromal tumor (very low risk). Immunohistochemical analysis revealed pMMR, and positive markers including CDX2, CK20, CD56, synaptophysin (Syn) and chromogranin A (CgA). The average Ki-67 labeling index was 80%. The stage was pT3N1M0. Postoperative detection of human peripheral blood circulating tumor cells (CTC) by multiple mRNA fluorescence *in situ* hybridization indicated that a total of 20 circulating tumor cells were detected, including 11 epithelial types, and 9 mixed types. We recommended postoperative adjuvant therapy for the patient. However, the patient’s postoperative condition was not good, so the time for postoperative adjuvant therapy was delayed.

Approximately 2 months after surgery, a CT scan revealed liver metastasis (Figure 1B&C) with portal vein thrombosis (Figure 1D). Subsequently, the PD-L1 immunohistochemistry (IHC) 22C3 pharmDx assay and immunofluorescence (IF) were used to characterize the tumor microenvironment (TME). The results of the PD-L1 IHC 22C3 assay showed that the tumor proportion score (TPS) was 5% and the combined positive score (CPS) was 10 (Figure 1G). However, the IF PD-L1 (E1L3N) assay showed TPS <1% and CPS <1. In addition, the composition of immune cells in the TME was determined by IF analysis, and the results (Figure 1H&I) showed CD3^+^, CD4^+^, and CD8^+^ T cell infiltration in the tumor parenchyma and stroma (5.09% vs 14.94%, 0.35% vs 0.52%, 0.66% vs 0.30%, respectively). Moreover, the antitumor M1-like tumor-associated macrophages (TAMs) were more dominant than the pro-tumor M2-like phenotype in the tumor parenchyma (0.8% vs 0.06%), reflecting a favorable anti-tumor immune response. In addition, we analyzed the genetic characteristics by whole exon sequencing (WES). A total of 8 tumor driver genes were detected (Table 1), the tumor mutational burden (TMB) was 1.09363, and the frequency of microsatellite instability (MSI) occurrence in somatic cells (total number of MSI sites present in somatic variation/total number of MSI sites present in the sample) was 3.09%.

**TABLE 1:** Somatic mutation (driver mutation) identified in this patient.

Gene	Chr	Position	Ref	Alt	Variant classification	Gene_Description
TP53	Chr17	7,577,018	C	A	Splice_Site_mutation	Tumor protein p53
SETX	Chr9	135,156,882	T	A	Missense_mutation	Senataxin
RB1	Chr13	48,951,052	A	T	Splice_Site_mutation	Retinoblastoma 1
CFH	Chr1	196,645,183	C	T	Missense_mutation	Complement factor H
ZNF638	Chr2	71,582,901	T	C	Missense_mutation	Zinc finger protein 638
LRP1B	Chr2	141,114,006	CA	C	Frameshift_deletion	Low-density lipoprotein receptor-related protein 1B
SETX	Chr9	135,156,881	C	CA	Frameshift_insertion	Senataxin
LRP1B	Chr2	141,114,004	C	CT	Frameshift_insertion	Low-density lipoprotein receptor-related protein 1B

Chr: Chromosome, Ref: Reference base, Alt: Alternate base.

Subsequently, the patient received etoposide (150mg, ivgtt, d1-3, q3w) and cisplatin (50mg, ivgtt, d1-3, q3w) combined with toripalimab (240mg, ivgtt, d1, q3w) treatment. According to the Response Evaluation Criteria in Solid Tumors version 1.1, efficacy was assessed as partial remission after three cycles of treatment. After six cycles, both CT and positron emission tomography/computed tomography (PET/CT) scans showed complete regression of tumor lesions (Figure 1E). In addition, the number of CTCs was significantly reduced compared with the pre-treatment level (Figure 1J). Subsequently, the patient was treated with a maintenance regimen of S1 combined with toripalimab in December 2022. The patient underwent a CT scan in March 2023, and no signs of a tumor were found. Additionally, no significant immune-related side effects (irAEs) were observed during treatment.

## Discussion

Gastrointestinal tumors with both exocrine and neuroendocrine components were first described by Cordier in 1924 (Jiang et al., 2023) and officially renamed MiNEN in the 2019 WHO classification system (Jiang et al., 2023). MiNEN consists of non-neuroendocrine components and neuroendocrine components, one of which must comprise at least 30% of the tumor. At present, the diagnosis of MiNEN is mainly based on immunohistochemical techniques and morphological characteristics. However, the differential diagnosis is difficult when both components are poorly differentiated, so the diagnosis of MiNEN remains challenging (Zhang et al., 2021). Due to its rarity, there is currently no standard treatment. Surgery remains by far the most important treatment option, and studies have shown that even patients with advanced disease may benefit from palliative primary tumor resection (Li et al., 2022). However, the recurrence rate after radical surgery is as high as 45.7% (Lin et al., 2021). In addition, stage T3-4 and lymph node metastasis are independent risk factors for distant recurrence, and the liver is the most common site for distant recurrence. For patients with advanced tumors that progress rapidly, chemotherapy is the preferred treatment.

In this work, the patient was diagnosed with poorly differentiated adenocarcinoma by preoperative biopsy, while the postoperative pathological diagnosis was MiNEN, suggesting that it is difficult to diagnose MiNEN by biopsy alone, and that it is recommended to evaluate pathological specimens after surgical removal of the entire tumor. Moreover, the TME characteristics of tumor-infiltrating lymphocytes (TILs) and PD-L1 positive expression suggest that this patient may benefit from immune checkpoint inhibitors (ICIs). The patient subsequently received treatment with chemotherapy in combination with toripalimab. Efficacy was assessed as CR after 6 cycles of treatment. To date, patients have a progression-free survival time of more than 10 months.

Notably, in this case, both IHC and IF were used to detect the expression of PD-L1, but the results were contradictory. In addition to considering the consistency of PD-L1 antibody clones, the discordant results may also be related to observer variability and tumor expression heterogeneity. The discordance between PD-L1 test results has been reported in previous studies, and such discrepancies may cause patients to miss opportunities to receive appropriate anti-PD-1/PD-L1 therapy (Yeong et al., 2020). Therefore, the analysis of multiple indicators is conducive to the more accurate identification of patients who may benefit from immunotherapy. In addition, the results of IF indicated intensive TILs in the tumor. As is well known, the main function of ICIs is to relieve immune suppression and normalize the function of immune cells, and the therapeutic effect is better for tumors with more infiltrated immune cells. Therefore, it is of great significance to analyze the composition of immune cells in tumors for screening potential beneficiaries of ICIs.

WES is a technique that is widely used clinically. With this technology, we can identify MSI, TMB and some gene mutations, which can help select patients suitable for ICIs. However, the results of WES in this patient did not provide definitive evidence for immunotherapy. In the future, in addition to optimizing sequencing technology, we will need more research to find valuable genes or mutations to guide clinical treatment.

In conclusion, this study is the first to report a patient with PD-L1 high expression gastric MiNEN, and the characteristics of the TME were consistent with adaptive immune resistance. The patient responded favorably to chemotherapy combined with toripalimab, achieving complete regression of the tumor lesion after six cycles of treatment, and no irAEs occurred during treatment. Therefore, this treatment strategy should be considered when treating these rare gastric MiNEN patients with high PD-L1 expression.

## Data Availability

The raw data supporting the conclusions of this article will be made available by the authors, without undue reservation.
